# Increasing dominant follicular proportion negatively associated with good clinical outcomes in normal ovarian responders using the depot GnRH agonist protocol: a large-sample retrospective analysis

**DOI:** 10.1186/s13048-022-00973-7

**Published:** 2022-04-13

**Authors:** Houming Su, Youhua Lai, Jie Li, Tingting Liao, Licheng Ji, Xinyao Hu, Kun Qian

**Affiliations:** Reproductive Medicine Center, Tongji Hospital, Tongji Medical College, Huazhong University of Science and Technology, Wuhan, China

**Keywords:** IVF/ICSI, Controlled ovarian hyperstimulation, HCG trigger time, Depot GnRH agonist protocol, Dominant follicular proportion, Clinical outcomes

## Abstract

**Background:**

Currently, there is no universal criteria for the trigger time of controlled ovarian hyperstimulation (COH), especially with the emerging depot GnRH agonist protocol. It is challenging to explore an indicator that is representative of target follicle cohort development as an alternative to the conventional approach of determining the trigger time based on a few leading follicles.

**Methods:**

This was a large-sample retrospective analysis. Between January 2016 and January 2020, 1,925 young normal ovarian responders who underwent their first in vitro fertilisation (IVF)/intracytoplasmic sperm injection (ICSI) cycle using the depot GnRH agonist protocol were included. They were divided into three groups based on the dominant follicular proportion (DFP, defined as the ratio of ≥ 18 mm dominant follicles/ ≥ 14 mm large follicles on the human chorionic gonadotropin (HCG) day; Group A: < 30%; Group B: 30%−60%; and Group C: ≥ 60%). The binary logistic regression and multivariate linear regression were used to assess whether the DFP was associated with clinical pregnancy, the number of frozen blastocysts, the blastocyst formation rate, and the low number of frozen blastocysts.

**Results:**

The logistic regression analysis showed that compared with Group A, the odds ratio (OR) for clinical pregnancy was 1.345 in Group B (*P* = 0.023), and there was no statistical difference between Group C and Group A (*P* = 0.216). The multivariate linear regression analysis showed that DFP was negatively associated with the number of frozen blastocysts (β ± SE: Group B vs. Group A = − 0.319 ± 0.115, *P* = 0.006; Group C vs. Group A = − 0.432 ± 0.154, *P* = 0.005) as well as the blastocyst formation rate (β ± SE: Group B vs. Group A = − 0.035 ± 0.016, *P* = 0.031; Group C vs. Group A = − 0.039 ± 0.021, *P* = 0.067). Furthermore, the OR for the low number of frozen blastocysts was 1.312 in Group B (*P* = 0.039) and 1.417 in Group C (*P* = 0.041) compared to Group A.

**Conclusions:**

For young normal ovarian responders using the depot GnRH agonist protocol, increasing DFP might reduce the developmental potential of oocytes and reduce the number of available blastocysts, and this might result in a lower cumulative pregnancy rate. However, further confirmation using strict prospective randomised controlled studies is required.

**Supplementary information:**

The online version contains supplementary material available at 10.1186/s13048-022-00973-7.

## Introduction

Controlled ovarian hyperstimulation (COH) is considered a key factor in the success of in vitro fertilisation (IVF)/intracytoplasmic sperm injection (ICSI) [1, 2] because it induces the development of multiple follicles to obtain as many high-quality oocytes as possible, thereby increasing the number of embryos available for transfer and ultimately increasing pregnancy rates. A crucial step during COH is to optimise the timing of the human chorionic gonadotropin (HCG) trigger to obtain better pregnancy outcomes [2, 3]. To date, there is no universal criteria for the optimal HCG trigger time [1, 2, 4, 5]. The common standard in most reproductive centres is to trigger when there exist at least three follicles ≥ 17 mm or at least two follicles ≥ 18 mm [1, 2, 6–8]. However, triggering is typically delayed in the expectation of obtaining more mature follicles in practice. Ectors et al. suggested that delaying the HCG trigger allows for larger follicle growth and may result in more mature oocytes, and this may have a positive impact on clinical outcomes [9]. However, several studies have shown that delayed oocyte retrieval does not increase the number of mature oocytes nor the pregnancy rate, but it may instead compromise embryo quality [8]. Confusingly, some studies have found that there is no difference in the oocyte maturation rate and the fertilisation rate between large and small follicles (10−14 mm; 15−19 mm; 20 + mm) [10]; (< 16 mm; ≥ 16 mm) [11]. These inconsistent findings might be due to differences in COH protocols, the definitions of follicle size, patient characteristics, insemination methods (IVF or ICSI), and endpoint metrics [1].

Currently, the available COH protocols are flexible and diverse. The depot GnRH agonist protocol is an emerging protocol that is widely used in clinical practice (especially in China). In this case, the pituitary is deeply desensitised by a long-acting GnRH agonist, and endogenous luteinizing hormone (LH) is profoundly suppressed (< 1 IU/L). Due to a lack of relevant studies, its optimal trigger time is unclear, and some reproductive centres still follow the trigger criteria of the short GnRH-agonist long protocol. However, the mechanism of action, endocrine hormones, and medication regimen differ greatly between the different COH protocols [4]. The choice of the same trigger time for the different COH protocols is controversial [1, 12]. Thus, evidence is still urgently required regarding the trigger time of the depot GnRH agonist protocol.

During COH, follicle growth is often asynchronous. Hence, we need to focus on the development of target follicle cohort, not just a few leading follicles [8]. The proportion of ≥ 17 mm follicles was used to assess the trigger time of the GnRH antagonist protocol, and this reflected the development of the follicle cohort and contributed to more high-quality oocytes and higher pregnancy rates [5]. In this study, dominant follicular proportion (DFP) was first proposed in the depot GnRH agonist protocol, which was the ratio of ≥ 18 mm follicles/ ≥ 14 mm follicles via transvaginal ultrasound on the HCG day (≥ 18 mm follicles defined as dominant follicles and ≥ 14 mm follicles defined as the large follicles). In addition, we analysed the relationship between the DFP and clinical outcomes, including the oocyte maturation rate, the normal fertilisation rate, the clinical pregnancy rate, the implantation rate, the number of blastocysts frozen, the blastocyst formation rate, and the low number of frozen blastocysts.

## Materials and methods

### Patient selection

This was a retrospective study. All of the eligible patients who underwent and completed their first IVF/ICSI cycle with the depot GnRH agonist protocol from January 2016 to January 2020 at the Reproductive Medicine Center of Tongji Hospital, affiliated with the Huazhong University of Science and Technology, were recruited.

The inclusion criteria were as follows: (1) it was the patient’s first IVF/ICSI fresh embryo transfer cycle, (2) the female with age < 35 years, (3) body mass index (BMI) ≤ 30 kg/m^2^, (4) antral follicle counting (AFC) ≥ 5, (5) anti-Müllerian hormone (AMH) ≥ 1.2 IU/L, (6) basal serum follicle-stimulating hormone (FSH) < 12 IU/L, and (7) 6−18 retrieved oocytes.

The exclusion criteria were as follows: (1) donor oocyte cycles/oocyte cryopreservation cycles, (2) the female had polycystic ovary syndrome; and (3) testicular sperm aspiration/testicular sperm extraction/ microsurgical epididymal sperm aspiration/percutaneous epididymal sperm aspiration cycles.

## Controlled ovarian stimulation protocols

For the depot GnRH agonist protocol, 3.75 mg of long-acting GnRH-a (Decapeptyl; Ferring, Saint-Prex, Switzerland) was administered subcutaneously on day two of the menstrual cycle. Pituitary suppression was evaluated 28 days after pituitary downregulation. The criteria for confirming the success of downregulation were as follows: follicle diameter < 5 mm, serum LH < 5 IU/L, serum estradiol (E2) < 50 pg/mL, and endometrial thickness < 5 mm. Then, COH was started using 75 to 300 IU/day of recombinant human FSH (Gonal-F; Merck-Serono, Geneva, Switzerland). When at least three leading follicles ≥ 17 mm or two leading follicles ≥ 18 mm were observed via a transvaginal ultrasound, a dose of 0.25 mg of recombinant HCG (Ovidrel; Merck-Serono, Geneva, Switzerland) was administered to trigger ovulation. After 36−37 h, oocyte retrieval was performed. In the IVF cycle, a short period of insemination was performed, and the oocytes were stripped four hours later. In the ICSI cycle, oocytes were stripped two to four hours after retrieval, and the MII stage oocytes were selected for single sperm injection. The maturation of the oocytes and the condition of the dipoles were observed under an inverted microscope after stripping. The criteria for fresh cycle transfer were: serum progesterone < 1.5 ng/mL [13], number of oocytes retrieved < 20, and serum E2 level < 7,000 pg/mL. The luteal phase was supported if the embryo transfer was performed. Fewer than two best-quality embryos were transferred or frozen on day three after oocyte retrieved, according to the protocol developed by Chinese legislation. The remaining embryos were cultured to days five or six to form blastocysts and then frozen for later transfer during the subsequent frozen embryo transfer cycles.

### Definition of the DFP levels and groups

The number of dominant follicles ≥ 18 mm and the number of large follicles ≥ 14 mm were counted and recorded in detail during the transvaginal ultrasound on the HCG day. The DFP was defined as ≥ 18 mm follicles/ ≥ 14 mm follicles. We established the groups as DFP < 30% (Group A), 30−60% (Group B), and ≥ 60% (Group C). Group A corresponded to at least two follicles ≥ 18 mm on the HCG day, which was the most common trigger reference.

### Clinical outcomes

The number of mature oocytes (MII) was counted four hours after the short period of IVF insemination or when the ICSI was performed. The oocyte maturation rate was the ratio of the MII/retrieved oocytes in both the IVF and ICSI cycles. Fertilisation was assessed 16 to 18 h after insemination. The presence of two pronucleus (2PN) was considered normal fertilisation. In addition, the normal fertilisation rate was the ratio of 2PN/retrieved oocytes (IVF) or 2PN/MII (ICSI). The blastocyst formation rate was the ratio of frozen blastocysts/embryos in a continuous culture. The low number of frozen blastocysts was defined as the number of frozen blastocysts ≤ 1 (i.e., less than 50% of the average number of frozen blastocysts). For the fresh cycle with embryo transfer on day three after oocyte retrieval, the clinical pregnancy rate and implantation rate were calculated. Clinical pregnancy was identified as the presence of an intrauterine gestational sac with fetal cardiac activity. The clinical pregnancy rate was the ratio of clinical pregnancies to embryo transfer cycles. The implantation rate was the ratio of implanted embryos to transferred embryos.

### Statistical analysis

The Pearson chi-square (χ2) test on the categorical variables and the analysis of variance (ANOVA) or Kruskall-Wallis H test on the continuous variables were performed as appropriate. Multivariate linear regression analyses were conducted for the predictive factors of the number of blastocysts frozen and the blastocyst formation rate. Moreover, binary logistic regression analyses were conducted for the predictive factors of clinical pregnancy and the low number of frozen blastocysts. The results are provided in terms of the 95% confidence intervals (CI) and the *P* values. A two tailed *P*-value of < 0.05 indicated statistical significance. All of the statistical analyses were conducted using the Statistical Package for Social Science version 25.0 (SPSS, Chicago, IL, USA).

## Results

A total of 1,925 consecutive IVF/ICSI cycles were included. As shown in Table 1, the baseline characteristics and ovarian response characteristics were matched in the three groups, and these included maternal age, BMI, basal FSH, dose of gonadotropin (Gn), and duration of Gn. There were no significant differences in terms of the oocyte maturation rate (IVF/ICSI), the normal fertilisation rate (IVF/ICSI), or the clinical pregnancy rate among the DFP groups. The implantation rate was highest in Group B (Group A 48.67%, Group B 57.57%, Group C 50.71%,* P* = 0.031). Interestingly, Group A had the highest number of frozen blastocysts (Group A 3.05 ± 2.35, Group B 2.60 ± 2.16, Group C 2.32 ± 2.12, *P* < 0.001) and the highest blastocyst formation rate (Group A 42.38% ± 26.59%, Group B 38.69% ± 27.16%, Group C 38.26% ± 28.27%, *P* = 0.047).

**Table 1:** Baseline characteristics and clinical outcomes

	Group A < 30%	Group B 30%-60%	Group C ≥ 60%	*P*
Cycles	398	1230	297	-
Duration of infertility(years)	3.21 ± 1.98	3.35 ± 2.14	2.97 ± 2.06	0.026
Maternal age (years)	29.17 ± 2.94	29.19 ± 2.84	29.13 ± 2.60	0.939
BMI (kg/m^2^)	22.24 ± 3.03	22.19 ± 3.09	22.42 ± 2.94	0.500
AFC	16.55 ± 6.42	16.39 ± 6.15	14.79 ± 5.01**	< 0.001
Basal FSH (mIU/ml)	7.27 ± 1.79	7.23 ± 1.69	7.32 ± 1.94	0.685
AMH (ng/ml)	6.47 ± 4.20	6.59 ± 4.00	5.72 ± 3.52**	0.003
Dose of Gn (IU)	2435.31 ± 962.72	2401.90 ± 855.15	2511.15 ± 811.93	0.149
Duration of Gn (days)	11.31 ± 2.01	11.25 ± 2.11	10.99 ± 1.84	0.087
Dose/Duration of Gn (IU/d)	214.10 ± 70.19	213.27 ± 63.17	228.88 ± 64.52**	0.001
E2 (HCG day, pg/ml)	2322.80 ± 1083.71	2289.81 ± 1047.77	2283.90 ± 1065.46	0.849
P (HCG day, ng/ml)	0.72 ± 0.34	0.79 ± 0.52*	0.82 ± 0.34**	0.010
≥ 14 mm follicles	11.92 ± 3.27	11.21 ± 3.17*	9.45 ± 2.95**	< 0.001
Oocytes retrieved	12.74 ± 3.22	12.22 ± 3.35*	11.59 ± 3.29**	< 0.001
Oocyte maturation rate (%)				
IVF	86.10 ± 14.39	88.17 ± 14.22	87.31 ± 14.57	0.101
ICSI	82.27 ± 14.87	80.59 ± 14.88	81.41 ± 13.05	0.553
Normal fertilisation rate (%)				
IVF	62.70 ± 16.99	63.26 ± 18.53	61.74 ± 19.25	0.525
ICSI	57.53 ± 19.46	57.78 ± 19.08	55.16 ± 17.80	0.573
Endometrial thickness (mm)	12.26 ± 2.58	12.30 ± 2.58	12.01 ± 2.38	0.223
Frozen blastocysts	3.05 ± 2.35	2.60 ± 2.16*	2.32 ± 2.12**	< 0.001
Blastocyst formation rate (%)	42.38 ± 26.59	38.69 ± 27.16*	38.26 ± 28.27**	0.047
Transfer one embryo				
Clinical pregnancy rate (%)	58.20 (188/323)	65.30 (510/781)	62.82 (98/156)	0.084
Transfer two embryos				
Clinical pregnancy rate (%)	68.00 (51/75)	73.94 (332/449)	67.38 (95/141)	0.232
Implantation rate (%)	48.67 (73/150)	57.57 (517/898) *	50.71 (143/282)	0.031

*BMI* = body mass index, *AFC = *antral follicle counting, *FSH = *follicle stimulating hormone, *IVF = *in vitro fertilisation, *ICSI* = intracytoplasmic sperm injection

* Group B versus Group A, *P* < 0.05; ** Group C versus Group A, *P* < 0.05

A binary logistic regression analysis was conducted to assess the relationship between the BMI, dose/duration of Gn, the number of oocytes retrieved, the number of embryos transferred, DFP, and clinical pregnancy (Table 2). The results showed that compared with Group A, the OR for clinical pregnancy was 1.345 in Group B (*P* = 0.023); however, there was no statistical difference between Group C and Group A (*P* = 0.216).

**Table 2:** Logistic regression analysis of clinical pregnancy

	OR	95% CI	*P*
BMI	0.935	0.904, 0.966	< 0.001
Dose/Duration of Gn	0.992	0.990, 0.993	< 0.001
Number of oocytes retrieved	0.950	0.920, 0.980	0.001
Number of embryos transferred			
2	1.469	1.180, 1.830	0.001
1	REF		
DFP			
Group A	REF		
Group B	1.345	1.041, 1.738	0.023
Group C	1.242	0.881, 1.751	0.216

*REF =* reference value. A *P*-value of < 0.05 indicated statistical significance

Multivariate linear regression analyses were performed to evaluate the relationship of infertility type, dose/duration of Gn, the number of oocytes retrieved, the fertilisation type, and DFP with the number of frozen blastocysts and the blastocyst formation rate. Surprisingly, the DFP was negatively associated with number of frozen blastocysts, as shown in Table 3 (β ± SE: Group B vs. Group A = − 0.319 ± 0.115, *P* = 0.006; Group C vs. Group A = − 0.432 ± 0.154, *P* = 0.005). In addition, the DFP was also negatively associated with the blastocyst formation rate, as shown in Table 4 (β ± SE: Group B vs. Group A = − 0.035 ± 0.016, *P* = 0.031; Group C vs. Group A = − 0.039 ± 0.021, *P* = 0.067).

**Table 3:** Multivariate linear regression analysis of the number of frozen blastocysts

	β ± Standard error	Standardized β	t	*P*
Infertility				
Secondary	0.194 ± 0.097	0.042	2.010	0.045
Primary	REF			
Dose/Duration of Gn	-0.003 ± 0.001	-0.078	-3.692	< 0.001
Number of oocytes retrieved	0.252 ± 0.014	0.380	18.051	< 0.001
Fertilisation				
ICSI	-0.513 ± 0.103	-0.104	-4.966	< 0.001
IVF	REF			
DFP				
Group A	REF			
Group B	-0.319 ± 0.115	-0.070	-2.769	0.006
Group C	-0.432 ± 0.154	-0.071	-2.798	0.005

*REF* = reference value. A *P*-value of < 0.05 indicated statistical significance

**Table 4:** Multivariate linear regression analysis of the blastocyst formation rate

	β ± Standard error	Standardized β	t	*P*
Infertility				
Secondary	0.027 ± 0.012	0.055	2.330	0.020
Primary	REF			
Dose/Duration of Gn	-0.0004 ± 0.0001	-0.095	-4.038	< 0.001
Number of oocytes retrieved	0.006 ± 0.002	0.071	3.029	0.002
Fertilisation				
ICSI	-0.027 ± 0.014	-0.045	-1.914	0.056
IVF	REF			
DFP				
Group A	REF			
Group B	-0.035 ± 0.016	-0.061	-2.160	0.031
Group C	-0.039 ± 0.021	-0.052	-1.833	0.067

*REF* = reference value. A *P*-value of < 0.05 indicated statistical significance

A binary logistic regression analysis was performed to evaluate the relationship of infertility type, dose/duration of Gn, number of oocytes retrieved, fertilisation type, and DFP with the low number of frozen blastocysts (Table 5). The results showed that compared with Group A, the OR for the low number of frozen blastocysts was 1.312 in Group B (95% CI = 1.014–1.698, *P* = 0.039) and 1.417 in Group C (95% CI = 1.014–1.979, *P* = 0.041).

**Table 5:** Logistic regression analysis of the low number of frozen blastocysts (≤ 1)

	OR	95% CI	*P* value
Infertility			
Secondary	0.768	0.621, 0.949	0.015
Primary	REF		
Dose/Duration of Gn	1.003	1.001, 1.005	< 0.001
Number of oocytes retrieved	0.832	0.806, 0.858	< 0.001
Fertilisation			
ICSI	1.464	1.175, 1.826	0.001
IVF	REF		
DFP			
Group A	REF		
Group B	1.312	1.014, 1.698	0.039
Group C	1.417	1.014, 1.979	0.041

The low number of frozen blastocysts = the number of frozen blastocysts ≤ 1; *REF *reference value. A *P*-value of < 0.05 indicated statistical significance

## Discussion

Currently, COH protocols are flexible and diverse. As the understanding of different protocols increases, we need to further focus on the effects of different endocrine hormonal environments on follicular cluster development and the clinical outcomes of IVF/ICSI. Since the introduction of the depot GnRH agonist protocol, pituitary desensitisation has typically been profound, and the endogenous LH level has been suppressed significantly (< 1.0 IU/L). LH plays an essential physiological role in follicular steroidogenesis, development, and maturation [14, 15]. There were no any data available in the published literature regarding when to administer the HCG trigger during the depot GnRH agonist protocol. Most reproductive centres still follow the same criteria for trigger timing when using this protocol as in the short GnRH-a long protocol. However, the traditional criteria for the HCG trigger time are not strict and remain controversial [2–5].

The previous views were that the follicular size was positively related to follicular maturity, fertilisation, and subsequent development [1, 2, 5, 16]. Oocytes derived from large follicles (14−21 mm; mean diameter: 19.1 ± 2.1 mm) seem to be more inclined to form high-quality embryos in the GnRH antagonist protocol [17]. Therefore, in practice, the HCG trigger is typically delayed with the aim of stimulating more follicle growth and obtaining as many mature oocytes as possible [8]. However, a previous study reported that oocytes in oversized follicles may decrease the quality and recovery of oocytes [5]. Our results showed no differences in the oocyte maturation rates or the normal fertilisation rates in the DFP groups. This was consistent with previous studies that demonstrated that enlarging the follicle size might not improve oocyte maturation or fertilisation [4, 18–20].

In our study, the clinical pregnancy rate and the implantation rate seemed to decrease as the DFP increased (Group B vs. Group C). Overgrowth of dominant follicles may lead to oocyte overmaturation, which in turn negatively affects the quality of oocytes and the clinical pregnancy outcome. One study was consistent with our results, and the high-quality embryo rate, pregnancy rate, and implantation rate were significantly higher in the low proportion group (≥ 18 mm follicle divided by the total number of follicles, low proportion: < 15%; middle proportion: 15–27%; high proportion: > 27% in the short GnRH-a long protocol) [8]. In two other randomised controlled trials of GnRH antagonist cycles, follicle size was enlarged by delaying HCG administration for one or two days after three follicles ≥ 17 mm. This corresponded to a decrease in the ongoing pregnancy rates in the delayed group [4, 18]. The availability of surplus embryos for freezing was lower when delaying two days compared with delaying one day, but this did not reach statistical significance [18]. Interestingly in our study, the number of frozen blastocysts and the blastocyst formation rate decreased significantly as the DFP increased (i.e., negative correlations). Furthermore, increasing the DFP was a risk factor for the low number of frozen blastocysts. These results revealed that the overgrowth of dominant follicles might lead to oocyte post-maturity, which in turn could have a negative effect on oocyte quality and ultimately lead to unsatisfying pregnancy outcomes [8, 21]. This phenomenon may be associated with an increased incidence of ultrastructural abnormalities in the oocytes, such as the appearance of a degenerated organelle-smooth endoplasmic reticulum (sER) [22]. Embryos with accumulated sER may have a lower rate of blastocyst formation and poorer pregnancy outcomes [23]. It is generally believed that sER accumulation is associated with high E2 levels on the HCG day and long-term Gn stimulation [22]. According to the results of clinical pregnancy and the available blastocysts, it is speculated that an excessive delay in the trigger might negatively affect the cumulative pregnancy rate. However, further prospective studies are required.

There are several strengths and limitations in our study. The most important innovation is the finding that the larger the DFP, the smaller the number of frozen blastocysts and blastocyst formation rate. In other words, increasing the DFP is a risk factor for the low number of frozen blastocysts and is also negatively associated with good clinical outcomes in general. Furthermore, our study was the first to analyse the depot GnRH agonist protocol in terms of the HCG trigger time. Finally, for the analysis of the DFP groupings and clinical outcomes, we used a large panel of data, established multiple linear regressions and binary logistic regressions, and conducted a thorough and comprehensive evaluation of these relationships. To a certain extent, this should provide useful information for clinical decision-making. The limitations are as follows. First, due to its retrospective nature, there may be some confounding bias. Second, we lacked the cumulative pregnancy rate results. For such a large sample, it was difficult to query and count the data. In addition, we screened standardised young normal ovarian responders. These results may be not applicable to older patients and low or high ovarian responders.

In summary, pursuing more and larger follicles during COH may not be beneficial to clinical outcomes. This practice might increase patient costs and time without increasing the oocyte maturation rate, normal fertilisation rate, or the number of embryos available for transfer. For patients who do not form any available blastocysts (surprisingly, these patients accounted for 1/5, according to our data), an early HCG trigger might be preferable to avoid diminishing the oocyte and embryo quality and consequently avoid poor IVF/ICSI outcomes.

## Conclusions

For normal ovarian responders during pituitary downregulation, increasing DFP on the HCG day might reduce the developmental potential of oocytes and decrease the available blastocysts, and this might result in a lower cumulative pregnancy rate. However, further confirmation using strict prospective randomised controlled studies is required.

## Supplementary Information

**Additional file 1.**

**Additional file 2.**

## Data Availability

The datasets used and/or analysed during the current study are available from the corresponding author upon reasonable request.
