# Supplementary material for: Increasing dominant follicular proportion negatively associated with good clinical outcomes in normal ovarian responders using the depot GnRH agonist protocol: a large-sample retrospective analysis
Source: J Ovarian Res. 2022 Apr 13;15:44. doi: 10.1186/s13048-022-00973-7 (PMC9006398; doi:10.1186/s13048-022-00973-7)
Supplement: Supplementary file 1 — Additional file 1. [file 13048_2022_973_MOESM1_ESM.pdf]

Certificate of English Language Editing

Manuscript Title:

Increasing dominant follicular proportion negatively associated with good clinical outcomes in normal ovarian responders using the depot GnRH agonist protocol: a large-sample retrospective analysis

Date of Revision:

March 16, 2022

Abstract:

Background: Currently, there is no universal criteria for the trigger time of controlled ovarian hyperstimulation (COH), especially with the emerging depot GnRH agonist protocol. It is challenging to explore an indicator that is representative of overall follicular population development as an alternative to the conventional approach of determining the trigger time based on a few leading follicles.

Methods: This was a large-sample retrospective analysis. Between January 2016 and January 2020, 1,925 young normal ovarian responders who underwent their first in vitro fertilisation (IVF)/intracytoplasmic sperm injection (ICSI) cycle using the depot GnRH agonist protocol were included. They were divided into three groups based on the dominant follicular proportion (DFP, defined as the ratio of ≥ 18 mm dominant follicles/ ≥ 14 mm large follicles on the human chorionic gonadotropin (HCG) day; Group A: < 30%; Group B: 30%[illegible]60%; and Group C: ≥ 60%). A binary logistic regression and multivariate linear regression were used to assess whether the DFP was associated with clinical pregnancy, the...

This document certifies that the manuscript listed above was copy edited for English language by LetPub, with regard to grammar, punctuation, spelling, and clarity. All of our language editors are native English speakers with long-term experience in editing scientific and technical manuscripts. We are committed to leveling the playing field for researchers whose native language is not English.

- Documents receiving this certification should be regarded as having undergone professional editorial revision for English language before submission. However, the authors may accept or reject LetPub's suggestions and changes at their own discretion and LetPub does not have editorial control over the submitted documents.
- The language quality of the submitted document is the sole responsibility of the submitting authors subject to those authors' adherence to LetPub's revisions and instruction. LetPub's provision of service does not constitute a guarantee or endorsement of the authors' work herein.
- Neither the research content nor the authors' intended meaning were altered in any way during the editing process.
- If you have any questions or concerns about this edited document, please contact us at support@letpub.com

LetPub
Your Manuscript Editor

LetPub is an author service brand owned and operated by Accdon LLC. Headquartered in the Boston area, we are a full-spectrum author services company with a large team of US-based certified language and scientific editors, ISO 17001 accredited translators, and professional scientific illustrators and animators. We advocate ethical publication practices and are an official member of the Committee on Publication Ethics (COPE).

For more information about our company, services, and partnership programs, please visit www.letpub.com.

© 2022 Accdon, LLC. All Rights Reserved. Tel: 1-781-202-9968 Email: info@accdon.com Address: 400 Fifth Ave, Suite 530, Waltham, MA 02451, United States
