# Supplementary material for: Increasing dominant follicular proportion negatively associated with good clinical outcomes in normal ovarian responders using the depot GnRH agonist protocol: a large-sample retrospective analysis
Source: J Ovarian Res. 2022 Apr 13;15:44. doi: 10.1186/s13048-022-00973-7 (PMC9006398; doi:10.1186/s13048-022-00973-7)
Supplement: Supplementary file 2 — Additional file 2. [file 13048_2022_973_MOESM2_ESM.docx]

**Manuscript JOVR-D-21-00414R1**

**Response to Reviewers**

**Dear editors,**

**Thank you for giving us the opportunity to submit a revised draft of the manuscript “Increasing dominant follicular proportion negatively associated with good clinical outcomes in normal ovarian responders using the depot GnRH agonist protocol: a large-sample retrospective analysis” for publication in the Journal of Ovarian Research. We appreciate the time and effort that you and the reviewers dedicated to providing feedback on our manuscript and are grateful for the insightful comments on and valuable improvements to our paper. We have incorporated most of the suggestions made by the reviewers. Those changes are underlighted within the manuscript. Please see below, in italic, for a point-by-point response to the reviewers’ comments and suggestions. All page numbers and line numbers refer to the revised manuscript file.**

**Reviewer #1:**

**1. The manuscript was not written in clear language which made it very difficult to read and understand. It should be be rewritten in simple, accurate and logical English for scientific publication purpose.**

*We appreciate this reviewer’s suggestion. And we have asked an expert mother tongue scientific writer to polish our paper (the certification is attached). Those changes are underlighted within the revised manuscript. For example, the abstract is modified as follows.*

***“Background:*** *Currently, there is no universal criteria for the trigger time of controlled ovarian hyperstimulation (COH), especially with the emerging depot GnRH agonist protocol. It is challenging to explore an indicator that is representative of target follicle cohort development as an alternative to the conventional approach of determining the trigger time based on a few leading follicles.*

***Methods:*** *This was a large-sample retrospective analysis. Between January 2016 and January 2020, 1,925 young normal ovarian responders who underwent their first in vitro fertilisation (IVF)/intracytoplasmic sperm injection (ICSI) cycle using the depot GnRH agonist protocol were included. They were divided into three groups based on the dominant follicular proportion (DFP, defined as the ratio of ≥ 18 mm dominant follicles/ ≥ 14 mm large follicles on the human chorionic gonadotropin (HCG) day; Group A: < 30%; Group B: 30%−60%; and Group C: ≥ 60%). The binary logistic regression and multivariate linear regression were used to assess whether the DFP was associated with clinical pregnancy, the number of frozen blastocysts, the blastocyst formation rate, and the low number of frozen blastocysts.*

***Results:*** *The logistics regression analysis showed that compared with Group A, the odds ratio (OR) for clinical pregnancy was 1.345 in Group B (P = 0.023), and there was no statistical difference between Group C and Group A (P = 0.216). The multivariate linear regression analysis showed that DFP was negatively associated with the number of frozen blastocysts (β ± SE: Group B vs Group A = −0.319 ± 0.115, P = 0.006; Group C vs Group A = −0.432 ± 0.154, P = 0.005) as well as the blastocyst formation rate (β ± SE: Group B vs Group A = −0.035 ± 0.016, P = 0.031; Group C vs Group A = −0.039 ± 0.021, P = 0.067). Furthermore, the OR for the low number of frozen blastocysts was 1.312 in Group B (P = 0.039) and 1.417 in Group C (P = 0.041) compared to Group A.*

***Conclusions:*** *For young normal ovarian responders using the depot GnRH agonist protocol, increasing DFP might reduce the developmental potential of oocytes and reduce the number of available blastocysts, and this might result in a lower cumulative pregnancy rate. However, further confirmation using strict prospective randomised controlled studies is required.”*

**2. The study repeatedly mentioned that the major concern of the study is to determine the "Triger time" in the targeting COH treatment in Abstract, Introduction, Results and Conclusion. But the team did not generate a clear conclusion to provide suggestion to clinicians with a "ideal trigger time".**

*We thank the reviewer for raising this important point. During COH, follicle growth is often asynchronous, we need to focus on the development of target follicle cohort, not just a few leading follicles. In this study DFP (≥18 mm follicles /≥14 mm follicles) included both the leading follicles and the large follicle cohort, and was considered a more valid and objective indicator. Group A (DFP < 30%) corresponded to two leading follicles ≥18 mm on HCG day, which was the most common trigger standard. Delayed triggering would result in an increase in DFP. However, increasing DFP might reduce the developmental potential of oocytes, reduce the number of available blastocysts and was a risk factor for low number of available blastocysts (Line 143-145, Page 7; Line 151-157, Page 7-8; Line 158-162, Page 8).*

**3. As my knowledge, the leading follicle size (biggest follicle among all follicles) on the hCG trigger day will be an important factor affect the cycle in some IVF cycles. Please consider to add the parameters to the study to evaluate its impacts.**

*We thank the reviewer for raising this important point. Follicle growth is asynchronous during COH, we aim to focus on the development of follicle cohort. In addition, the number of frozen blastocysts and cumulative pregnancy rate are associated with the large follicle cohort, not just the leading follicle. DFP (≥18 mm follicles /≥14 mm follicles) included both the leading follicles and the large follicle cohort. And Group A (DFP < 30%) corresponded to two leading follicles ≥18 mm on HCG day, which was the most common trigger standard.*

**4. As the study included both ICSI and IVF cycles, please indicated if ICSI cycles included any severe male factor cases like TESE/MESA/TESA cycles.**

*We thank the reviewer for having raised the point. The testicular sperm aspiration/testicular sperm extraction/ microsurgical epididymal sperm aspiration/percutaneous epididymal sperm aspiration (TESA/TESE/MESA/PESA) cycles were excluded in the study, and we have now highlighted this in“Material and methods” section (Line 85-87, Page 5).*

**5. I see the Maturation rate evaluation in table 1 included "conventional IVF cycle". It is very difficult to understand how to evaluate Maturation Rate in conventional IVF as no oocyte stripping was used in it. Please provide more information about method.**

*We would like to thank the reviewer for this feedback and now added a more precise description of oocyte stripping and the evaluation of oocyte maturation rate in IVF in “Material and methods” section (Line 97-98, and Line 100-101, Page 5; Line 115-116, Page 6).*

**6. I am sure in most ICSI cycles, there are immature eggs retrieved including M1 and GV. I would suggest the study includes the number/ratio of M1 and/or GV oocytes as the maturity is clearly associated to follicular sizes.**

*We would like to thank the reviewer for these further valuable insights. Several studies have shown that oocyte maturation rate is not related to follicle size (Line 47-51, Page 3). There was also no statistical difference in oocyte maturation rate between DFP groups in our study (Table 1, Page 16). Furthermore, we found that oocyte maturation rate was not associated with DFP in the multiple linear regression model (β ± SE: Group B vs Group A = -0.002±0.005, P = 0.727; Group C vs Group A = -0.004±0.006, P = 0.510; unpublished data). In addition, the risk of low oocyte maturation rate was not associated with DFP in the logistic regression model (OR: Group B vs Group A = 1.121, P = 0.633; Group C vs Group A = 1.368, P = 0.230; unpublished data).* *The main aim of our study was to derive the relationship between DFP and clinical outcomes. Only MII stage oocytes were selected for single sperm injection in ICSI, so we did not include the number/ratio of M1 and/or GV oocytes in this large-scale study.*

**7. It is necessary to add completed description to "Material and methods" including Oocyte maturation evaluation protocol in both IVF and ICSI cycles, Embryo culture protocol, Embryo transfer protocol (Day 3 or Day 5 transfer) et al.**

*We would like to thank the reviewers for his/her time and effort reviewing our manuscript and we now provide more in-depth descriptions of oocyte maturation evaluation protocol in both IVF and ICSI cycles (Line 97-101, Page 5; Line 115-116, Page 6), embryo culture protocol (Line 105-107, Page 5), embryo transfer protocol (Line 103-105, Page 5) in "Material and methods".*

**Associate Editor comments**

**The study is very interesting but it needs extensive revision by expert mother tongue scientific writer. The Authors should improve their paper following the Reviewer' comments.**

*We are grateful for the editor’s positive evaluation and constructive criticism of our work. We have asked an expert mother tongue scientific writer to polish our paper. Those changes are underlighted within the revised manuscript. And we have improved the paper following the Reviewer’ comments in the revised manuscript point to point.*

**In my opinion, they should underline in the title and in the text that the findings regard potentially good responder patients who received a GnRH agonist protocol.**

*We are grateful to the editor, and we now underline in the title and in the text that the findings regard normal ovarian responders using the depot GnRH agonist protocol (Title, Page 1; Line 14-16, and Line 30, Page 1; Line 217-219, Page 10; Line 227, Page 11).*

**They should clarify if excluded or included (and in this case the proportion) women with PCOS.**

*We are grateful for the editor’s suggestion, and now clarify that women with PCOS were excluded in this study (Line 85, Page 5).*

**I did not understand the evaluation of follicles less than 5mm as criteria to start ovulation induction.**

*We would like to thank the editor for this feedback and now added a more precise description in “Materials and methods” (Line 91-97, Page 5). The criteria for confirming downregulation in depot GnRH agonist protocol were as follows: follicle diameter <5 mm, serum luteinizing hormone (LH) <5 mIU/ml, serum estradiol (E2) <50 pg/mL, and endometrial thickness < 5 mm. COH was started after the downregulation. When at least three leading follicles ≥ 17 mm or two leading follicles ≥ 18 mm were observed, rHCG was administered to trigger ovulation.*

**I suggest to avoid the etrm "prolonged". In several papers this term includes GnRH agonist treatment for more than 3 months in patients with endometriosis.**

*We would like to thank the editor for drawing attention to the ambiguous use of the term “prolonged”, which we now rename “depot GnRH agonist protocol”.*

**Please detail better in the abstract section the "dominant follicular proportions (DFP)".**

*We thank the editor for having raised the point, in response to which we now detail the abstract of "dominant follicular proportions (DFP)" (Line 16-17, Page 1).*
